# Affective and Motivational Factors Mediate the Relation between Math Skills and Use of Math in Everyday Life

**DOI:** 10.3389/fpsyg.2016.00513

**Published:** 2016-04-19

**Authors:** Brenda R. J. Jansen, Eva A. Schmitz, Han L. J. van der Maas

**Affiliations:** ^1^Department of Developmental Psychology, University of AmsterdamAmsterdam, Netherlands; ^2^ABC Amsterdam Brain, and Cognition, University of AmsterdamAmsterdam, Netherlands; ^3^Yield, Research Institute of Child Development and Education, University of AmsterdamAmsterdam, Netherlands; ^4^Department of Psychological Methods, University of AmsterdamAmsterdam, Netherlands

**Keywords:** gender, math performance, math anxiety, perceived math competence, numeracy

## Abstract

This study focused on the use of math in everyday life (the propensity to recognize and solve quantitative issues in real life situations). Data from a Dutch nation-wide research on math among adults (*N* = 521) were used to investigate the question whether math anxiety and perceived math competence mediated the relationship between math skills and use of math in everyday life, taken gender differences into account. Results showed that women reported higher math anxiety, lower perceived math competence, and lower use of math in everyday life, compared to men. Women's skills were estimated at a lower level than men's. For both women and men, higher skills were associated with higher perceived math competence, which in turn was associated with more use of math in everyday life. Only for women, math anxiety also mediated the relation between math skills and use of math in everyday life.

## Introduction

Math skills are important for functioning in everyday life as well as in various professions. Everyday life is full of challenges that demand math-related activities. Keeping a budget for example concerns most adults, both at large scale (e.g., in a household) and small scale (e.g., when shopping). It requires an overview and weighing of financial incomes and costs. Another example is planning, crucial for both adolescents and adults, demanding the reading of time tables or the assessment of activities' lengths in order to arrive or finish in time. As a final example, many individuals deal with the estimation of quantities when cooking or decorating their house. These situations are just a few examples but demonstrate the importance of using number knowledge, mathematical operations, and knowledge of math-related concepts like time. Nowadays, technology more and more provides devices to face these challenges, which often remove the need for mental calculations. However, also when using technological devices, mental calculations and estimations are crucial for a hunch of the outcome of for example a route planner or to check whether a discount is really beneficial. Reyna and Brainerd (2007) emphasize the relevance of mathematics skills for making decisions in everyday life, and note that a large number of adults in the USA do not possess the math skills “to handle the quantitative tasks of everyday life” (Reyna and Brainerd, 2007, p. 156). They also acknowledge that skills only do not suffice to handle these tasks. Here, we use data from a nation-wide research on math in the Netherlands, which offer the opportunity to investigate whether both math skills and emotional and motivational factors (math anxiety and perceived competence) are related to the use of mathematics in everyday life. Use of math in everyday life is defined as the propensity to recognize and solve quantitative issues in real life situations.

One affective factor that might play a role in the relationship between math skills and use of math in everyday life is math anxiety. Math anxiety can be conceived of as a performance-based anxiety, sharing important symptoms with other performance-based anxieties, such as social anxiety (Hopko et al., 2002; Ashcraft et al., 2007), which are experienced in situations that demand performance or when anticipating performance (see also Lyons and Beilock, 2012). Math anxiety refers to the persistent feelings of tension, apprehension and excessive fear in situations that require solving math problems in both ordinary life and academic situations (Beilock and Ramirez, 2011; Wu et al., 2014). Math anxiety has been shown to have a mutual negative relationship with math performance, often expressed in a correlation of around −0.3 (e.g., Hembree, 1990; Ma, 1999). Low math performance may cause the development of math anxiety (e.g., Hopko et al., 2002). The other way around, math anxiety may cause low math performance when, for example, anxiety-characteristic worries and arousal decrease performance (e.g., Ashcraft and Krause, 2007; Ashcraft and Moore, 2009). An alternative way in which math anxiety may cause low math performance is when avoidance inhibits the exercise of skills. Avoidance of math occurs when students rush through math work or exams, postpone math homework, drop math-related courses in high school, use heuristics instead of cognitive reflection, and limit use of math in everyday life (Hembree, 1990; Ashcraft, 2002; Morsanyi et al., 2014). The present study sets out to study this association, between the degree of math anxiety and the avoidance of math in everyday life.

Additionally, gender differences are of specific relevance, as numerous studies show that women report higher levels of math anxiety than men (Hembree, 1990; Meece et al., 1990; Miller and Bichsel, 2004; Bonnot and Croizet, 2007; Marsh et al., 2008; Devine et al., 2012), although other studies show only small gender differences (Chinn, 2009) or no gender differences at all (Chiu and Henry, 1990; Ma, 1999; Ho et al., 2000; Ma and Xu, 2004; Birgin et al., 2010; Erturan and Jansen, 2015). A gender difference in math anxiety may relate to the lower female participation in professions in science, technology, engineering and mathematics (STEM; Bureau of Labor Statistics, US Department of Labor, 2014; www.cbs.nl). Both females' elevated level of report of math anxiety and their lagging representation in technical professions cannot easily be explained by skill differences. Gender differences in mathematics performance fluctuate with the measurements used and the country under study (Else-Quest et al., 2010), but range from girls outperforming boys in math grades (Pomerantz et al., 2002), to no gender differences (Miller and Bichsel, 2004; Devine et al., 2012) and small male advantages (Liu and Wilson, 2009).

An important factor in the realization of gender differences in math anxiety seems to be whether the assessment concerns state or trait math anxiety. Trait anxiety concerns individuals' beliefs on their anxiety, whereas state anxiety concerns momentary emotions (Robinson and Clore, 2002). Goetz et al. (2013) assessed both state and trait math anxiety and showed that individuals' reports on trait math anxiety were often higher than those on state math anxiety. In the present study, we assess individuals' trait math anxiety, which has been shown to relate to math performance (e.g., Hembree, 1990), and avoidance of math-related activities (Chinn, 2009).

Our first research question concerns the association between math anxiety and the use of math in everyday life, taking math skills and gender differences into account. We hypothesize that there is a positive relation between math skills and use of math in everyday life that is however mediated by math anxiety, in the sense that higher skills are negatively related to math anxiety, which is again negatively related to use of math in everyday life. The relations between math skills, use of math in everyday life and math anxiety are investigated for women and men separately because the relationship between math skills and math anxiety is expected to be stronger for women than for men (Devine et al., 2012; Erturan and Jansen, 2015; but see Hembree, 1990; Meece et al., 1990; Ma and Xu, 2004; Miller and Bichsel, 2004).

A second factor that might play a role in the relationship between math skills and the actual use of math in daily life is an individuals' perceived competence of performing math. Various concepts of self-beliefs exist and definitions sometimes overlap. Central in concepts like self-efficacy and perceived competence is a person's perception of his/her competence, sometimes in relation to peers (Harter, 1982; Jansen et al., 2013). Self-beliefs about math are related to career interest in math and science (O'Brien et al., 1999) as well as to mathematics anxiety (Meece et al., 1990). Control-value theory (Pekrun, 2006) states a negative causal relation between perceived control of success and anxiety. Anxiety may result from both the expectation of being unsuccessful in a given situation and the valuing of success in the situation. Indeed, this relationship is supported empirically for the domain of math (Bieg et al., 2013). The mutual relation between self-belief and mathematics performance is established as well (Marsh et al., 2005; Liu, 2009; Erturan and Jansen, 2015). Here, we focus on perceived math competence: A person's feeling of being competent to successfully accomplish math tasks. A high confidence in one's math competence may ease the use of mathematics in everyday life. Reports of females' lower self-beliefs concerning math, compared to males', are more numerous (Meece et al., 1990; Pomerantz et al., 2002; Else-Quest et al., 2010; Goetz et al., 2013) although reports of similar levels of perceived math competence have been reported as well (Jansen et al., 2013; Erturan and Jansen, 2015).

Our second research question centers on the role of perceived math competence in the use of math in everyday life, next to math anxiety, and taking into account math skills and gender differences. We hypothesize that the relation between math skills and use of math in everyday life is also mediated by perceived math competence, in the sense that math skills are positively related to perceived math competence, which is again positively related to use of math in everyday life. The possible mediating effects of math anxiety and perceived math competence are included simultaneously, in one model. The present data allow for investigating whether math performance has an impact on math anxiety through perceived math competence, as might be derived from control-value theory (Pekrun, 2006). However, our interest is on the relation between perceived math competence and use of math in everyday life, a concept which is only scarcely studied, taking into account relations between math anxiety, perceived math competence, and math performance. Again, the relation is investigated separately for men and women.

### The present study

The present study is conducted as part of a nation-wide research on math in the Netherlands. A large scale data collection was conducted concerning different facets of mathematics, for various studies on mathematics of different researchers. For the present study, data on emotional and motivational factors as well as the use of math in everyday life and math skills have been investigated. Data collection was online, which allowed participants to fill in the tests and questionnaires in their own time, in a familiar environment. The collected data offer the opportunity to investigate our research question, that is, whether math anxiety and perceived math competence mediate the relationship between math skills and use of mathematics in everyday life. Regarding gender differences, we hypothesize that (1a) women report higher levels of mathematics anxiety than men; (1b) women's math skills are equal to those of men; (1c) women report lower levels of perceived math competence than men. Regarding the relation between math skills, use of math in everyday life, math anxiety, and perceived math competence, we hypothesize that (2a) the relation between math skills and use of math in everyday life is positive but (2b) is mediated by math anxiety, in the sense that math skills are negatively related to math anxiety, which is negatively related to use of math in everyday life. Finally, we hypothesize that (2c) the relation between math skills and use of math in everyday life is also mediated by perceived math competence, in the sense that math skills are positively related to perceived math competence, which is again positively related to the use of math in everyday life. The relation between math skills and use of math in everyday life, possibly mediated by math anxiety and perceived math competence, is investigated separately for men and women.

## Methods

### Participants

The Grand National Research on Math is an initiative of the Netherlands Organization for Scientific Research (NWO), and two Dutch broadcasters. Participants responded to calls in a Dutch television program on popular science and on the Internet to fill out questionnaires on math and solve math problems on a central website of the Grand National Research. Different tasks and questionnaires were presented on the website. Participants were free to choose what they were interested to do on the website and thus which parts to complete. A total of 1066 individuals filled in the questionnaire on math in everyday life. From this sample, 556 participants also filled in the questionnaires on math anxiety and perceived math competence and finished at least one session of the addition game in Math Garden (see below). Data from 20 participants were excluded because they were younger than 18 years old. Additionally, data from 15 participants, who had followed primary school outside the Netherlands, were excluded. The final sample consisted of 521 participants (59% females). The average age of the participants was 45.72 years (*SD* = 14.68; range: 18.54–79.14 years).

The upper panel of Table 1 shows the number of women and men, by level of highest completed education. The sample had a relatively high level of education, compared to the general population in the Netherlands. A chi-square test demonstrated that highest completed education and gender were not independent, χ${}_{\left(6\right)}^{2}=19$.76, *p* = 0.003. Relatively more men than women had finished higher secondary education. However, this category contained only a minority of the participants (10%) and it is not very likely that the skewed distribution in this category would cause a gender difference in math skills in the present sample. The lower panel of Table 1 shows the number of women and men by profession, in descending order of total frequency. Only the seven most frequently named professions are shown. Unemployed participants and students did not answer this question. Gender distribution differed across professions, χ${}_{\left(8\right)}^{2}=72$.64, *p* < 0.001. Relatively more women worked in care and welfare, whereas relatively more men worked in ICT and construction and engineering professions, reflecting Dutch societal differences (www.cbs.nl).

**Table 1:** **Numbers of women and men in the current sample, by level of highest completed education and by profession**.

	**Females (% of females)**	**Males (% of males)**	**Total (% of total sample)**
**LEVEL OF HIGHEST COMPLETED EDUCATION**			
PhD	12 (4%)	9 (4%)	21 (4%)
Master's degree	71 (23%)	48 (22%)	119 (23%)
Bachelor's degree	108 (35%)	63 (29%)	171 (33%)
Higher sec. educ.	16 (5%)	36 (17%)	52 (10%)
Vocational educ.	43 (14%)	23 (11%)	66 (13%)
Intermediate sec. educ. or lower	10 (3%)	6 (3%)	16 (3%)
No response	47 (15%)	29 (14%)	76 (15%)
**PROFESSION**			
Education	48 (16%)	20 (9%)	68 (13%)
Care and welfare	51 (17%)	14 (7%)	65 (13%)
ICT	8 (3%)	38 (18%)	46 (9%)
Trade and hospitality	8 (3%)	13 (6%)	21 (4%)
Science	14 (5%)	6 (3%)	20 (4%)
Economy and finance	6 (2%)	12 (6%)	18 (4%)
Construction and engineering	3 (1%)	14 (7%)	17 (3%)
Other	40 (13%)	16 (7%)	56 (11%)
Students	47 (15%)	29 (14%)	76 (15%)
No income from profession	82 (27%)	52 (24%)	134 (26%)
Total	307	214	521

### Material

#### Math anxiety

A measurement of math anxiety was obtained by administering the Dutch translation of the Math Anxiety Scale for Children (MASC; Chiu and Henry, 1990; Dutch translation was reported in Jansen et al., 2013). The overarching national research was set up to include both children and adults. Hence, a questionnaire was selected that could serve all age groups. The MASC could be administered to students, reporting their current math anxiety, and to adults, who were asked to report on their math anxiety in retrospect. A child questionnaire can be relevant for adults because many math-related experiences were at school, which is a period that most adults can vividly remember. Both positive and negative feelings around math often arise at school.

The MASC consisted of 23 statements, for example “Listening to the teacher in a math class” and “Waiting to get a math test returned in which you expect to do well.” Participants rated their anxiety on a four-point scale, ranging from 1 (“not nervous”) to 4 (“very nervous”). Scores ranged from 23 to 92, with a higher score indicating a higher level of (retrospective) math anxiety.

#### Perceived math competence

Perceived math competence was assessed using an adaptation of the scale Perceived Math Competence (Jansen et al., 2013), which was an extension of the Perceived Competence Scale for Children (Harter, 1982; Dutch translation by Veerman et al., 1997). Adaptation concerned the answer format of the scale. The scale consisted of six statements. Example statements were “It takes me long to solve math problems” and “I am struggling with math.” Statements were relevant for both children and adults. Participants indicated the extent to which each statement applied to them, using a four-point scale, ranging from 1 (“does not apply to me at all”) to 4 (“fully applies to me”).

#### Math skills

An approximation of math skills was obtained using a customized version of Math Garden. Math Garden is a computer-adaptive web-based practice and monitoring system for math (Klinkenberg et al., 2011). In this customized version, four math games were presented. Here, we focused on the addition game. Correlations between the addition game and the other games (mental arithmetic, series, 24-game) were high. A session of the addition game consisted of 15 sequentially presented addition problems, like 3 + 4, 234 + 48, and 234.78 + 32.98. Each addition problem was presented with six answer options, of which only one was correct and participants had 20 s to select the correct answer. A response was followed by highlighting the correct response alternative. Correct responses were rewarded, whereas errors were penalized. Penalty and reward of responses were linearly related to response time: Fast errors were more severely penalized than slow errors, whereas fast, correct responses were higher rewarded than slow correct responses (Maris and Van der Maas, 2012).

Selection of problems was adaptive, meaning that a more difficult problem was presented after a correct response and an easier problem after an error. Problem difficulties were extracted from Math Garden (Klinkenberg et al., 2011). Based on both response time and accuracy, each participant's ability was rated on a scale that ranged from approximately −10 to +10, although the end points were in principle infinite. A person's ability was adjusted upwards in case of a correct response and adjusted downwards in case of an incorrect response. Degree of adjustment depended on both speed and difficulty of the presented math problem (Klinkenberg et al., 2011).

#### Everyday life

Table 2 shows the questionnaire that was developed for the present study to assess use of math in everyday life, i.e., the propensity to recognize and solve quantitative issues in real life situations. The questionnaire consisted of 18 situations of possible applications of math in everyday life and 2 questions on the number of math-related activities that were employed in free time or in performing a profession. Each of the 18 situations was presented in an unfinished sentence, together with multiple question-specific complements to choose from. An example of a situation was “When paying in a shop…,” with complements “I do not check the amount of money returned,” “I look at the cashier to know the amount to be returned,” and “I know the exact amount to be returned” (see Table 2 for statements; see Appendix in Supplementary Materials for complements). Participants selected the complement that applied most to them. Two points were assigned to a complement that was judged on forehand to be associated with performing math, without any aids; one point was assigned to a complement that was associated with estimation or using a tool or device; no points were assigned to remaining complements. The response “inapplicable” was recorded as missing. The two additional questions on engagement in math-related activities in free-time or in a profession had multiple options to choose from (see items 19 and 20 in Table 2). Participants could indicate their engagement in up to 2 math-related activities in free time (score: 0–2) and in up to 4 math-related job activities (score 0–4). The total score on the everyday life questionnaire could range from 0 to 42, with a higher score corresponding to increased math-related activities in everyday life.

**Table 2:** **Questionnaire on use of math in everyday life**.

**Unfinished statement**	**Factor loadings**				**Mean score (SD) (range 0–2)**
	**1**	**2**	**3**	**4**	
2. If there is a discount on a product	**0.46**	0.01	0.10	0.24	1.4 (0.50)
4. When paying in a shop	**0.50**	−0.15	0.21	**0.35**	1.6 (0.69)
7. When adding 68 and 178	**0.72**	0.25	−0.02	−0.06	1.9 (0.33)
8. When adding three monetary amounts	**0.79**	0.05	0.02	−0.07	1.7 (0.46)
9. If the clock is adjusted, I know if I have to get up sooner or later because	0.24	**0.55**	0.06	−0.03	1.5 (0.59)
10. I'll find out the number of days in each month	0.18	**0.65**	−0.21	0.01	1.5 (0.50)
12. If I'm in a different time zone and want to know the time in the country of departure	−0.04	**0.44**	0.28	0.04	1.7 (0.60)
17. I locate the south at daytime	−0.13	**0.63**	0.08	0.10	1.7 (0.69)
5. If I pay with paper money	0.27	−0.13	**0.43**	0.13	1.8 (0.58)
15. If I'm going to paint a wall	0.11	0.10	**0.61**	−0.04	1.5 (0.56)
16. If I cook soup for eight guests, but the recipe is for six	0.04	0.01	**0.71**	−0.22	1.6 (0.50)
1. When doing errands	0.18	−0.06	−0.14	**0.68**	0.8 (0.44)
6. When receiving the bill in a restaurant	−0.10	0.19	0.00	**0.70**	0.9 (0.56)
11. If I travel to a new destination by car and need to be there on time	−^1^	−^1^	−^1^	−^1^	0.8 (0.39)
13. If I travel to an unknown destination by bike or car I determine my route	−^1^	−^1^	−^1^	−^1^	0.9 (0.26)
14. If I travel to an unknown destination by public transport, I determine my route	−^1^	−^1^	−^1^	−^1^	1.0 (0.14)
20. In my spare time (multiple answers possible)	−^1^	−^1^	−^1^	−^1^	0.9 (0.75)
3. When I fill out my tax forms	−^2^	−^2^	−^2^	−^2^	1.4 (0.90)
19. For my profession (multiple answers possible)	−^2^	−^2^	−^2^	−^2^	1.3 (1.36)^3^

*Items are arranged by factor. Factor loadings higher than 0.30 are printed in bold*.

1 Item was not included in Principal Component Analysis because of low inter-item correlations;

2 *Item was not included in Principal Component Analysis because Cronbach's alpha decreased if item was deleted*.

3 *Scores can range from 0 to 4*.

### Procedure

The Grand National Research on Math was performed under the responsibility of the Netherlands Organization for Scientific Research (NWO), and two Dutch broadcasters. The research was announced in a television show on popular science. Viewers were notified of the possibility to voluntarily participate in the online research. Visitors of the website were first explained the privacy policy of the research. Participants were informed that participation was anonymous, that results were not traceable to individuals and that data were used for scientific purposes only, respecting the Data Protection Act. Participants had the possibility to enter their e-mail address in case they would like to be informed of their personal scores, but e-mail addresses were not used in data processing. No personal information was used for scientific research. Participants had complete control of continuing or terminating their participation because the researcher was not present during the research and participants could leave the website whenever they wanted. Material did not relate to medical issues, did not include a screening procedure and chance incidents were not possible. There was no deception. Discomfort due to participation was unexpected. For Math Garden, the Ethical Committee of the University of Amsterdam approved of the procedure of passive consent.

Upon their first visit of the website, participants received a personal identity number. Participants answered general questions on demographic information. Next, participants were free to participate in any of the studies on math. The present measures were reached by using three links: one for the questionnaires on math anxiety and perceived math competence, one for Math Garden, and one for the questionnaire on use of math in everyday life. Participants were free to choose order and timing of responding to the measures and any order was allowed. Data on the order of responding to the measures were not logged, making it impossible to test whether filling out one measure (e.g., the questionnaire on math anxiety and perceived math competence) has affected performance on a different measure (e.g., Math Garden).

## Results

### Use of math in everyday life questionnaire: reliability and factor structure

Reliability and factor structure of the questionnaire on math in everyday life were investigated first because the questionnaire was newly developed. Data from all 1066 participants who responded to the questionnaire were included. Items 11, 13, 14, and 20 were excluded from further analyses because scores on these items had low inter-item correlations (average correlation was below 0.05). Calculations of Cronbach's alpha if items were deleted pointed to the additional exclusion of items 3 and 19. Cronbach's alpha was α = 0.687 for the remaining 14 items.

A Principal Component Analysis, using direct oblimin rotation, resulted in the extraction of four factors with an eigenvalue higher than 1. Together, the factors explained 45.8% of the variance. Loadings for the four factors are presented in Table 2. Items that referred to an interest in mental arithmetic loaded highest on the first factor, which was coined “Mental Arithmetic.” The second factor seemed to concern knowledge of math-related facts like how to locate the south at day-time and was coined “Math-related Facts.” Items on use of math in daily situations like converting the amount of ingredients of a recipe loaded highest on the third factor, which was coined “Practical Math.” Items that referred to keeping a budget (doing errands, a restaurant bill) loaded high on the fourth factor, coined “Budget.” Note that factors Mental arithmetic and Practical math fitted the definition of use of math in everyday life best. Internal consistency of an aggregate of the 8 items that loaded highest on these 2 factors (> 0.4) was α = 0.628. Further analyses were performed with both the total sum scores of the 14 items (Total use everyday life) and the sum score on the 8 items that had high loadings on factors Mental Arithmetic and Practical Math (“Mental and practical math use”).

### Investigating gender differences in math anxiety, perceived math competence, use of math in daily life, and addition skills

The hypotheses on (the absence of) gender differences in math anxiety, perceived math competence, and math skills were investigated next. We studied gender differences in the use of math in everyday life exploratory because no hypothesis was formulated for this domain. Mean scores by gender for math anxiety, perceived math competence, addition skill ratings and use of math in everyday life are presented in Table 3. A Multivariate Analysis of Variance (MANOVA) with math anxiety and perceived math competence, addition skill and use of math in daily life as dependent variables and gender as the independent variable showed a significant main effect of gender, *F*_(5, 515)_ = 18.75, *p* < 0.001, η^2^ = 0.154. *Post-hoc* univariate tests showed that gender differences were observed for all variables. As expected, females reported higher levels of math anxiety and lower levels of perceived math competence than males. Females' estimated addition skills were lower than males', which was unexpected. Finally, females reported lower use of math in everyday life, compared to males.

**Table 3:** **Descriptive statistics for math anxiety, perceived math competence, ratings of addition skill, and use of math in everyday life**.

	**Mean (*****SD*****)**		**Univariate test**	
	**Females**	**Males**	***F*_(1, 519)_**	**η^2^**
Math anxiety	38.12 (14.55)	29.73 (7.67)	59.56^*^	0.103
Perceived math competence	17.41 (4.99)	21.04 (3.20)	87.91^*^	0.145
Ratings of addition skills	6.86 (1.46)	7.25 (1.47)	9.06^*^	0.017
Use of math in daily life: total	20.22 (3.78)	22.13 (2.85)	39.34^*^	0.070
Use of math in daily life: mental and practical math use	12.45 (2.54)	13.49 (1.97)	25.17^*^	0.046

* *p < 0.001*.

Note that the effect size for the gender difference in addition skills was much lower than that for all other variables. Exploratory, we studied whether gender would explain additional variance in math anxiety and perceived math competence, when already taking into account addition skills. This was tested in a MANOVA with math anxiety and perceived math competence as dependent variables and gender, addition skills, and the interaction between gender and addition skills as independent variables. All main effects and the interaction effect were significant in the MANOVA.

The main effect of skill was significant, implying that for individuals with lower skills math anxiety was higher *F*_(1, 517)_ = 42.02, *p* < 0.001, η^2^ = 0.075, and perceived math competence was lower, *F*_(1, 517)_ = 62.66, *p* < 0.001, η^2^ = 0.108. The main effect of gender indicated higher math anxiety and lower perceived math competence scores for women compared to men (see Table 3). The interaction effect between gender and addition skills was significant for math anxiety, *F*_(1, 517)_ = 24.75, *p* < 0.001, η^2^ = 0.046, and perceived math competence, *F*_(1, 517)_ = 62.66, *p* < 0.001, η^2^ = 0.108. It was investigated by performing multigroup regression analyses with math anxiety/perceived math competence as the dependent variable, skills as the independent variable, and gender as group variable. As expected, given the interaction effect, estimating different values for the relation between skills and math anxiety for men and women improved the model significantly, χ${}_{\left(1\right)}^{2}=28.95$, *p* < 0.001 for math anxiety; χ${}_{\left(1\right)}^{2}=8$.428, *p* = 0.004 for perceived math competence. Concerning math anxiety, the relation with skills was not significant for men (*B* = −0.522, *p* = 0.141), but significant for women (*B* = −3.965, *p* < 0.001). Concerning perceived math competence, the relation with skills was weaker for men (*B* = 0.642, *p* < 0.001) than for women (*B* = 1.311, *p* < 0.001).

In sum, the results supported hypotheses 1a and 1c, that females were associated with higher math anxiety and lower perceived math competence than males. Hypothesis 1b, that gender differences would be absent in addition skills, was not supported as females' estimated addition skills were lower than males'. However, these gender differences in skills did not fully explain the gender differences in reported math anxiety and perceived math competence. Independent of skills, females reported higher math anxiety and lower perceived math competence.

### Mediation effects of affective and motivational factors in the relation between skills and use of math in everyday life

Next, it was investigated whether the relation between skills and use of math in everyday life was positive and mediated by both math anxiety and perceived math competence. Multigroup analyses were performed, with gender as group variable. First, a set of hierarchical regression analyses was conducted to investigate the predictive value of skills on use of math in everyday life (Step 1) and the possible added predictive value of math anxiety and perceived math competence (Step 2). In both step 1 and step 2, it was tested whether the estimates of the predictor(s) could be restricted to be equal across genders.

Table 4 summarizes the results of the hierarchical regression analysis, by gender. In step 1, the model improved significantly when estimating the relation between skills and use of math in everyday life for men and women separately, χ${}_{\left(1\right)}^{2}=11.083$, *p* = 0.001, compared to a model in which this estimate was restricted to be equal across genders. Although positive for both, the relation was stronger for women than for men (see Table 4). In step 2, restricting the parameter estimating the predictive value of perceived math competence did not deteriorate the model significantly, χ${}_{\left(1\right)}^{2}=2.127$, *p* = 0.145. Restricting the parameter estimating the predictive value of math anxiety however did deteriorate the model significantly, χ${}_{\left(1\right)}^{2}=7.676$, *p* = 0.006. Hence, Table 4 shows the estimates of the multigroup model with gender-specific relations between skills as well as math anxiety and use of math in everyday life, and a general relation between perceived math competence and use of math in everyday life. For men, only the positive relation between perceived competence and use of math in daily life was significant. For women, addition skills as well as math anxiety and perceived math competence significantly predicted use of math in everyday life, in the expected directions.

**Table 4:** **Hierarchical multigroup regression analyses predicting use of math in everyday life by addition skills, math anxiety and perceived math competence, with gender as group variable**.

	**B**	**SE B**
**MALES**		
**Step 1: model including total effect of addition skills**		
Addition skills	0.271^*^ (0.113)	0.131 (0.091)
**Step 2: model including direct effect of addition skills**		
Addition skills	0.107 (0.006)	0.126 (0.088)
Math anxiety	−0.009 (0.006)	0.026 (0.018)
Perceived math competence	0.248^***^ (0.172^***^)^a^	0.044 (0.031)
**FEMALES**		
**Step 1: model including total effect of addition skills**		
Addition skills	0.909^***^ (0.521^***^)	0.138 (0.095)
**Step 2: model including direct effect of addition skills**		
Addition skills	0.292^*^ (0.115)	0.127 (0.089)
Math anxiety	−0.073^***^ (−0.045^***^)	0.017 (0.012)
Perceived math competence	0.248^***^ (0.172^***^)^a^	0.044 (0.031)

*Estimates and statistics for model with Mental and practical math use as outcome variable in brackets*.

a *Restricted to be equal across genders*.

* p < 0.05;

*** *p < 0.001*.

The same model selections were made when using scores on Mental and practical math use only (see Table 4 for estimates): The relation between addition skills and use of math in everyday life was gender-specific, χ${}_{\left(1\right)}^{2}=9.500$, *p* = 0.002, just like the relation between math anxiety and use of math in everyday life, χ${}_{\left(1\right)}^{2}=10.802$, *p* = 0.001, but not the relation between perceived math competence and use of math in everyday life, χ${}_{\left(1\right)}^{2}=3.591$, *p* = 0.058. For men, again only perceived math competence was related to use of math in everyday life. For women, there was again a significant negative relation between math anxiety and use of math in everyday life and a significant positive relation between perceived math competence and use of math in everyday life. The relation between addition skills and use of math in everyday life was not significant anymore.

**Figure 1:**
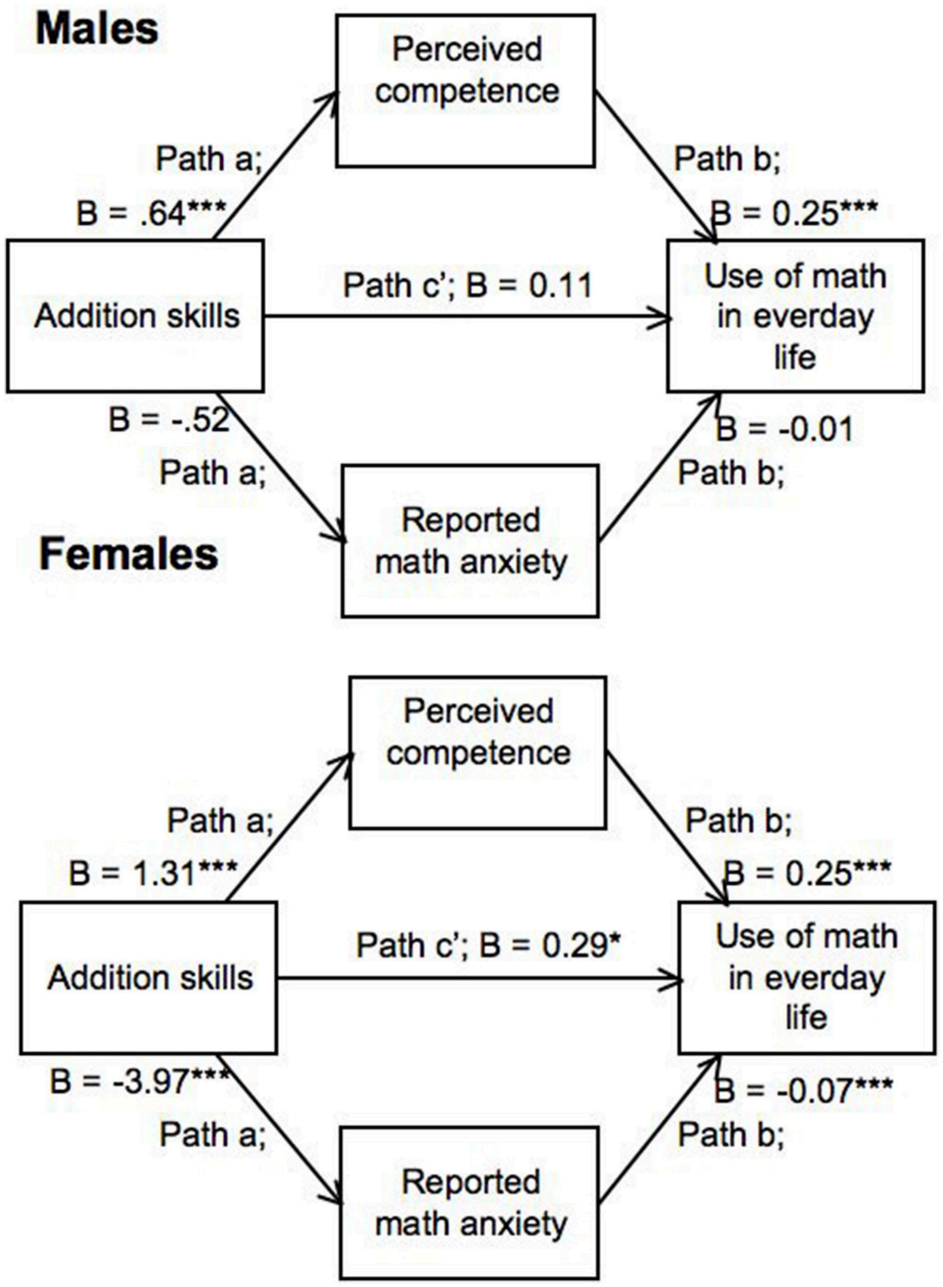
**Multigroup mediation model with relation between perceived math competence and use of math in everyday life restricted to be equal across genders**. All other parameters were estimated freely. ^*^p < 0.05; ^***^p < 0.001.

Next, multigroup mediation analyses were performed. A model with all parameters restricted to be equal across genders deteriorated the model significantly, χ${}_{\left(5\right)}^{2}=47.53$, *p* < 0.001, compared to a model where all parameters were estimated freely. The results of the hierarchical regression models suggested that the parameter that reflected the relation between perceived math competence and use of math in everyday life could be restricted to be equal across genders and this indeed did not deteriorate the model significantly, χ${}_{\left(1\right)}^{2}=2.13$, *p* = 0.094. This multigroup mediation model is shown in Figure 1. For men only the indirect path through perceived math competence, and not math anxiety, had significant relations. The indirect effect of perceived math competence was indeed significant for men (bootstrapped confidence interval: 0.08–0.27; determined using scripts by Selig and Preacher, 2008), supporting the hypothesis that perceived math competence mediated the relationship between addition skills and use of math in everyday life for men. For women, indirect paths through both math anxiety and perceived math competence showed significant relations. Both indirect effects turned out to be significant for women (bootstrapped confidence interval for math anxiety: 0.14–0.45; bootstrapped confidence interval for perceived math competence: 0.08–0.26).

Using scores on Mental and practical use only, model selection deviated slightly, resulting in the selection of the saturated model, where all parameters were estimated freely for men and women. For men, again only the indirect effect of perceived math competence was significant and for women again the indirect effects of both perceived math competence and math anxiety were significant. Interpretations of indirect effects were highly similar to the interpretations of the model when using the total score on the questionnaire for use of math in everyday life.

In sum, results supported hypothesis 2a that math skills (estimated with an addition task) were positively related to the use of math in everyday life for both men and women. For men, the relation was indirect, through the level of perceived math competence: Higher addition skills were related to higher perceived math competence, which was related to a higher use of math in everyday life, which matches expectations that follow from hypothesis 2c. For men, math anxiety however did not mediate the relation between skills and use of math in everyday life, which was contrary to expectations following hypothesis 2b. The absence of an effect of math anxiety is probably due to the very low reported levels of math anxiety by men in this sample (*M* = 29 with a possible range of 23–92). For women, high addition skills were related to an elevated level of perceived math competence, which was related to higher use of math in everyday life. Also, high addition skills were related to a lower level of math anxiety, and math anxiety was negatively related to use of math in everyday life. Hence, math anxiety and perceived math competence mediated the relation between math skills and use of math in everyday life for women, which matches expectations that follow from hypotheses 2b and 2c. It should however be noted that these correlational data provide the estimation of various mediation models. Indeed, estimating a mediation model with math anxiety as the outcome variable, math skills as the independent variable and use of math in everyday life as the mediating variable, resulted in the estimation of a significant mediation effect for women. Hence, drawing conclusions on causal relations is impossible using correlational data. The fact that various mediation models were possible (for women) does show the interrelatedness of math skills, math anxiety, perceived math competence, and use of math in everyday life.

## Discussion

In everyday life, mathematical thinking may benefit important choices, concerning for example medical and financial issues (Reyna and Brainerd, 2007). However, mathematical thinking might be hampered in various ways. In the current study, it was investigated whether math skills as well as affective (math anxiety) and motivational (perceived math competence) factors were related to men's and women's use of math in everyday life. The study was part of the Grand National Research on Math in the Netherlands and depended on voluntary registration of participants, which resulted in a sample size of over 500 adults. Gender differences in all measures were tested first. Results supported the hypotheses that women would report higher math anxiety and lower perceived math competence than men. Women also reported a lower use of math in everyday life. Unexpectedly, women's skills were estimated at a lower level than men's. Concerning the relationships, math skills and use of math in everyday life were positively related, as expected. For both women and men, the level of perceived math competence mediated the relation: Higher skills were associated with a higher sense of competence, which in turn was associated with more use of math in everyday life. Only for women, math anxiety also mediated the relation between math skills and use of math in everyday life: higher math skills were associated with lower math anxiety, which was related to a higher use of math in everyday life.

Females' higher level of reported math anxiety and lower level of perceived math competence, compared to males', is consistent with the majority of results of previous studies on gender differences in math anxiety (e.g., Hembree, 1990) and self-beliefs concerning math (e.g., Else-Quest et al., 2010; Cvencek et al., 2011). The gender gap may vary as a result of the sample characteristics (age, educational level, country, culture, and profession). In our sample, there was a higher percentage of men, compared to women, in technical professions, which reflects the underrepresentation of women in the science, technology, engineering and mathematics (STEM) professions in the Netherlands (www.cbs.nl). The relatively high percentage of males in technical professions might explain part of the gender gap found in this study. At least two explanations are possible. Either males in our sample were more technically skilled and had more technical interests than females in our sample, resulting in more technical jobs and possibly also reflected in higher math skills and use of math in everyday life. Hence, jobs and gender might be a confound. An alternative explanation would be that men indeed perform higher on the type of math test administrated in the current study. Higher skills might independently or dependently lead to lower math anxiety, higher perceived performance and more use of math in everyday life. It is striking that a gender gap in affective and motivational factors also exists in the current high-educated sample of adults. Note that also in general males tend to report lower levels of anxiety (e.g., Dyrbye et al., 2006) and higher levels of confidence (but see Britner and Pajares, 2006).

Females' lower estimate of addition skill, compared to males', was unexpected. The effect size of the difference was small, smaller than that of the gender differences regarding math anxiety and perceived math competence. The small effect size of the gender difference in math skills is in line with the literature, which is undecided and shows both female and male advantages on mathematics assessments. Situational differences may influence the direction of the advantage. Pressure and time limit may lower females' performance, in spite of an advantage in the classroom (Pomerantz et al., 2002). In the current study, the assessment was performed in a familiar, self-chosen environment, mostly in the participant's home. Estimates of ability were communicated to the participant only and had no consequences. These circumstances might reduce a possible gender difference in estimated ability. However, response time was limited, participants received accuracy feedback on each item, were rewarded for correct responses and penalized for mistakes and their estimated ability level was communicated to them. These aspects might increase a gender gap in estimated ability, in favor of males. In sum, although the assessment was set up as an assessment of addition skill, it might have been perceived of as a test of performance. It is unclear whether the gender difference should be perceived of as a male advantage of skill or of test-taking ability. Unknown is whether gender stereotypes about math played a role in the home situation. It has been found that these become activated in situations, resulting in more poorly performance of female (Spencer et al., 1999). Finally, the sample may have been biased if primarily those men who were confident of their math abilities chose to participate. Apart from these explanations for the gender gap in math skills, it should be noted that the difference was small. The modesty of the difference in skills however makes the larger gender difference in math anxiety and perceived math competence even more interesting: Despite only a small disadvantage in skills, women report higher math anxiety and lower perceived math competence than men.

Ashcraft et al. (2007) and Hopko et al. (2002) stress the importance of exercising skills to reach high performance. The observed relations in the present study may be interpreted as an illustration of this process and suggest that those who are weak at math should be provided with additional exercise because their weak skills may prevent them from using math in everyday life, missing out the required exercise. Moreover, Ashcraft et al. (2007) and Hopko et al. (2002) note that performance-based anxieties, like math anxiety, can hinder the exercise of skills. Indeed, in the current study, weak skills were associated with higher math anxiety, raising an extra barrier for practice. A downward spiral, linking skills, anxiety, exercise, and performance may emerge. Possibly, this is also reflected in the lower skills of women as they do not use it as often as men and also have professions more distant from technical jobs. However, note that data in the current study were correlational. Although the assessment of use of math in everyday life was related to both skills and math anxiety as well as perceived math competence, this does not imply that (experimentally) changing one of these factors would cause a change in any of the other factors.

Note that the most common professions in the present study were those in education, care, and welfare. In both types of professions, use of math is essential. Beilock et al. (2010) already showed the significance of teachers' own math anxiety for the development of their pupils' math skills. In medical professions, numeracy is essential as well, for example in calculating doses (e.g., McMullan et al., 2012). The present study shows the relevance of developing math skills as well as positive affect and feelings of competence for use of math in everyday life.

The current study is not without limitations. First, a proxy of math skills was used, using a computer-adaptive addition test. The selection of the addition test was based on high correlations with other math tests, but it remains an estimate, using time limits, automation of math facts, in only one domain. Second, the math anxiety questionnaire was based on school situations. As the initial aim of the study was to include child participants as well as adults, a children's questionnaire was used. Hence, participants were asked to fill in the questionnaire retrospectively. During the study, it turned out that participation from individuals under 18 years was low and in hindsight, an adult questionnaire might have been more appropriate. Replication with an adult math anxiety questionnaire is therefore desired. Even though, the correlation between the math anxiety questionnaire and estimated addition skills was comparable to what is reported in the literature and the selection of instruments for math anxiety and math skills seems justified. It would be interesting to study whether these correlations would hold using a questionnaire assessing state math anxiety instead of trait math anxiety. Goetz et al. (2013) showed important differences in the relation between math performance and math anxiety using either a trait or a state math anxiety assessment. Third, the questionnaire on the use of math in everyday life was developed from scratch for the current study. A challenge when developing such a questionnaire is to include only those situations that are applicable to all respondents. Although everyday life is full of math-related situations, these differ from person to person. Those responsible for a family face different challenges than for example students. Also, elderly people increasingly deal with medical situations and decisions and might use technology in a different way or may even lack any technological devices. In the current questionnaire, we started off with a range of situations. Statistical analysis showed that some questions were unrelated to the majority of the questions. Some subjects were relevant for only a small number of people. Also, in hindsight, some questions were more related to common knowledge and to keeping a budget than to the propensity to recognize and solve quantitative issues in real life situations. However, psychometric analyses detected these questions and the present questionnaire seems a good starting point. It can be improved by adding questions on the use of math when making medical and financial decisions, taking into account individual differences in everyday life. Moreover, technology is rapidly improving and people will adapt their use of math to the available technologies. For example, anticipating on the amount of change by looking for coins may not be so relevant in a world of digital payments. It should be considered from situation to situation whether full reliance on technological devices is possible or that mathematical thinking is still required to evaluate the outcomes of the device. Also, more exclusive answer options might be needed to cover the full range of individual differences in dealing with the situations described. Fourth, the present sample is self-selected and conclusions may be specific to this sample. The present data show that the current participants were relatively high-educated. Moreover, participants voluntarily visited the website of the Grand National Research on Math and it is very likely that they appreciated doing math. Participants could avoid the math skills test but only those who did take the test were included in the sample of the present study. Participants in the present sample may conceive of themselves as quite competent in math and less math-anxious than the general population. This hypothesis can only be tested in a replication study in a more general population. Importantly, the results on the gender gaps in math anxiety and perceived math competence and on the relationship between math anxiety and math skills are consistent with the majority of the results reported in the literature. The final and most critical drawback of the current study is its correlational nature. It is tempting to conclude that math skills cause math anxiety and/or the use of math in everyday life. However, all measurements were assessed under the same conditions, at the same time, without any manipulations and conclusions on causal relationships are impossible.

In sum, the present study supports the idea of a vicious circle linking skills, affective and motivational factors and use of math in everyday life, which has not been reported earlier in the literature. Individuals with high math skills use math more frequently in everyday life and are also more confident of their math abilities. For women, math anxiety is negatively related to using math in everyday life and to math skills. Use of math in everyday life, skills, affective and motivational factors may strengthen and mutually influence each other.

## Author contributions

All authors listed, have made substantial, direct and intellectual contribution to the work, and approved it for publication.

## Funding

Data for this study were collected in The Grand National Research on Math, which is an initiative of the Netherlands Organization for Scientific Research (NWO), and Dutch broadcasters NTR and VPRO.

### Conflict of interest statement

HV is scientific director of Math Garden, which was used to assess math skills. The other authors declare that the research was conducted in the absence of any commercial or financial relationships that could be construed as a potential conflict of interest.
