# Supplementary material for: Affective and Motivational Factors Mediate the Relation between Math Skills and Use of Math in Everyday Life
Source: Front Psychol. 2016 Apr 19;7:513. doi: 10.3389/fpsyg.2016.00513 (PMC4835718; doi:10.3389/fpsyg.2016.00513)
Supplement: Supplementary file 1 [file DataSheet1.docx]

Appendix. Questionnaire on use of math in everyday life

| Unfinished statement | | Complements (score between brackets) | % of responses |
| --- | --- | --- | --- |
| 1. When doing errands | | no idea of total (0) | 22% |
|  |  | estimate total (1) | 77% |
|  |  | know exact total (2) | 2% |
| 2. If there is a discount on a product | | estimate new price (1) | 55% |
|  |  | calculate new price (2) | 43% |
|  |  | use calculator for new price (1) | 2% |
| 3. When I fill out my tax forms | | independently (2) | 66% |
|  |  | leave to a professional (0) | 26% |
|  |  | inapplicable (missing) | 8% |
| 4. When paying in a shop | | do not check amount of money returned (0) | 12% |
|  |  | look at cashier for amount to be returned (1) | 14% |
|  |  | know exact amount to be returned (2) | 74% |
| 5. If I pay with paper money | | immediately look for change to ease returning money (2) | 85% |
|  |  | hand requested change (1) | 8% |
|  |  | never pay cash (0) | 8% |
| 6. When receiving the bill in a restaurant | | assume it is correct (0) | 20% |
|  |  | compare to estimate (1) | 68% |
|  |  | check thoroughly (2) | 12% |
| 7. When adding 68 and 178 | | by heart (2) | 87% |
|  |  | paper-and-pencil (1) | 8% |
|  |  | calculator (1) | 5% |
|  |  | use application e.g., Excel (1) | 1% |
| 8. When adding three monetary amounts | | by heart (2) | 71% |
|  |  | paper-and-pencil (1) | 20% |
|  |  | calculator (1) | 9% |
|  |  | use application e.g., Excel (1) | 1% |
| 9. If the clock is adjusted, I know if I have to get up sooner or later because | | look at others (0) | 5% |
|  |  | look up (1) | 13% |
|  |  | mnemonic (1) | 27% |
|  |  | by heart (2) | 56% |
| 10. I'll find out the number of days in each month | | by heart (2) | 53% |
|  |  | mnemonic (1) | 41% |
|  |  | look up (1) | 6% |
| 11. If I travel to a new destination by car and need to be there on time | | leave well in advance (0) | 17% |
|  |  | estimate departure time (1) | 30% |
|  |  | route planner for determining departure time (1) | 47% |
|  | | not applicable (missing) | 7% |
| 12. If I'm in a different time zone and want to know the time in the country of departure | | ask (0) | 6% |
|  |  | leave clock on time of departure country (1) | 7% |
|  |  | phone (1) | 15% |
|  |  | calculate (2) | 71% |
| 13. If I travel to an unknown destination by bike or car I determine my route | intuition or ask (0) | 7% |  |
|  | map (1) | 46% |  |
|  | navigation system or route planner (1) | 46% |  |
|  | inapplicable (missing) | 2% |  |
| 14. If I travel to an unknown destination by public transport, I determine my route | intuition or ask (0) | 2% |  |
|  | information leaflets (1) | 6% |  |
|  | Internet (1) / | 90% |  |
|  | inapplicable (missing) | 2% |  |
| 15. If I'm going to paint a wall | | ask for required number of cans (1) | 18% |
|  |  | estimate required number of cans (1) | 26% |
|  |  | calculate required number of cans (2) | 54% |
|  |  | buy a few cans of paint to start (0) | 3% |
| 16. If I cook soup for eight guests,  but the recipe is for six | | bit more of each ingredient (1) | 35% |
|  |  | make twice as much (1) | 5% |
|  |  | original recipe, give everyone less (0) | 1% |
|  |  | calculate required quantities of ingredients (2) | 60% |
| 17. I locate the south at daytime | | look at sun (2) | 81% |
|  |  | compass (2) | 5% |
|  |  | don't know (0) | 14% |
| 18. In the selection of a telephone subscription | | calculate costs of various subscriptions (2) | 52% |
|  |  | compare overall monthly costs of various subscriptions (1) | 33% |
|  |  | look at overall monthly costs of desired subscription (0) | 15% |
|  |  | inapplicable (missing) | 0% |
| 19. For my profession (multiple answers possible) | | use charts (1), programming (1),  study numerical information (1),  calculate (1) | - |
| 20. In my spare time (multiple answers possible) | | solve puzzles (1), mental exercises (1) | - |
|  |  |  |  |

*Notes.* Complements are abbreviated to save space.
